# Supplementary material for: Barriers to and impacts of hepatitis C treatment among people who inject drugs in Kenya: A qualitative study
Source: PLOS Glob Public Health. 2025 Jan 16;5(1):e0003284. doi: 10.1371/journal.pgph.0003284 (PMC11737709; doi:10.1371/journal.pgph.0003284)
Supplement: Attachment — Submitted filename: Response to Reviewers.docx [file pgph.0003284.s002.docx]

Dear Editors,

We have revised our manuscript per the reviewers’ recommendations, as outlined below. We thank the reviewers for their feedback. We feel these changes have improved the manuscript and we appreciate the opportunity to public our research in PLoS Global Public Health.

Sincerely,

The Authors

**Academic Editor Guillaume Fontaine’s Comments**

Thank you for submitting your work for our consideration. Enclosed you will find the peer reviewer reports. While there is interest in your paper, some areas require revision. Reviewer 1 has noted that the results section needs better alignment with the study’s aims. The study intends to explore treatment experiences and risk factors, yet the results primarily focus on barriers to HCV treatment and the impact of HCV treatment on individuals’ lifestyles and risk-taking behaviors, without addressing the personal experiences of receiving treatment. It would be beneficial to revise the title and aim of the paper to address this. Reviewer 2 pointed out that the study engaged few women, and further elaboration on the potential reasons and implications is necessary. This aspect should also be acknowledged in the study’s limitations. Additionally, while the study is guided by Rhodes’ Risk Environment Framework, few details are provided on this framework, and it is not referenced in the discussion to contextualize the findings. I recommend expanding on this framework and integrating it into your discussion. Finally, please provide the completed COREQ checklist as supplementary material.

**Reviewer #1’s Comments**

Thanks for the opportunity to review this manuscript. This study explored the experiences of PWID with Hepatitis C treatment and the changes in their risk-taking behaviors after treatment in Kenya. The authors applied robust methods and provided valuable insights into some of the barriers to treatment which have the potential to be used for designing future interventions. Below, please see my comments and suggestions.

Methods

1. Study Sample – The study participants were recruited among those who received treatment in the parent study. Could you please clarify whether the HCV treatment procedures/practices and follow ups in the parent study were different from the usual practices (i.e., how HCV treatment is normally offered at those centers)? (Please discuss this in Methods section). Is there any concern that experiences of those who received treatment via the parent study might be different from those who are treated via other routes (usual practices)? Please discuss its implications and how it can affect study findings. (Please discuss this in discussion).

**Response:** We have updated this part of the Methods section to note that the treatment procedures were carried out in accordance with standard treatment practices and followed national guidelines. We’ve noted in the limitations section that our sample may not be representative of all PWID seeking or undergoing HCV treatment due to population differences/sample bias, but did not include further comment on any differences given that the treatment procedures are the same.

2. Please provide more details on your sample size; how did you decide on the number needed? How many individuals did you ask to participate and how many refused to participate?

**Response:** We have added additional details on our sample size. We aimed to reach as many participants as possible until saturation was reached. No one who was asked refused to participate. One transcript was not included in the analysis as data on the participant was missing/incomplete.

Results

3. I suggest removing the age and gender of participants from identifiers after quotes and simply assign a number to them. I am worried that participants could be identified using age and gender, especially since they are frequenters of harm reduction centers.

**Response:** We have removed the ages and genders from the quote identifiers; they now only include the study number and region (Coast or Nairobi).

4. I suggest using “Environmental constraints/stressors and competing priorities” instead of the theme “Financial constraints,” because in addition to financial problems, you also discuss difficulty travelling to the treatment center, affording food, and managing other priorities.

**Response:** Thank you for your suggestion. We agree that this wording would better reflect the participants’ experiences and responses and have changed the theme.

5. Please add (in the results of in the methods) that all the participants received treatment for free. Also, in your discussion, please explain whether HCV treatment is covered in Kenya and how that could impact the generalizability of your findings to those who receive treatment through usual practices.

**Response:** Thank you for this suggestion. We have noted in the methods section that treatment was provided for free, and discussed potential ways in which the cost of treatment may affect treatment access/impacts. We have also explained that generalizability may be impacted by cost differences in the limitations section.

6. Line 204 – “In addition to the financial and geographic barriers to attending treatment sessions, participants discussed the challenges of navigating substance use recovery, stigma, and the role of family support (or lack thereof) that arose during HCV treatment” fits better under “HCV-related stigma.”

**Response:** We do agree that this quote and its discussion of family support/stigma related better to the stigma theme. However, we decided to remove the quote entirely rather than moving it, as the stigma section focused more on general community stigma and awareness and attitudes from other PWID, rather than familial relationships. We felt that removing the quote would better maintain the flow and cohesion of the results section.

7. As mentioned by the authors, the study aims to understand treatment experiences and risk factors of PWID. However, the results do not discuss individuals’ experiences of receiving treatment itself, for example did they experience any side effects, how was the interaction with the staff and healthcare providers, feelings about treatment… Instead, the results focus on barriers to HCV treatment and impact of HCV treatment on individuals’ lifestyle/risk-taking behaviors. I believe the title and aim of the study should reflect these results. I suggest modifying the title and aim slightly to something similar to this: “Barriers to/challenges of (receiving) Hepatitis C treatment and the impact of treatment on risk-taking behaviors among people who inject drugs in Kenya: A qualitative study”

**Response:** We have revised the title and aims described in the introduction section to better reflect the focus and findings.

Discussion

8. Line 327 – “Our data from PWID who received HCV treatment in two regions in Kenya provide valuable insights into patients’ perceptions about the HCV treatment process.” I find this sentence a bit misleading as the treatment process is not discussed in the results. As mentioned previously, I suggest this: “Our data from PWID who received HCV treatment in two regions in Kenya provide valuable insights into patients’ perceived barriers to HCV treatment…”

**Response:** We have revised this sentence to better reflect the focus of the paper and conclusions.

9. Line 332 – “They also provide support for the feasibility and benefit of treating PWID,” although the findings support the benefits of treating PWID, they do not show whether treatment is feasible. I would remove “feasibility.”

**Response:** We have removed feasibility per your suggestion.

10. In discussion, you mention the effect of the pandemic on individuals’ experiences with HCV treatment. Please report this finding in your results as well and provide some quotes from participants if possible.

**Response:** While we appreciate this comment, we believe the reviewer may have misunderstood this paragraph of the discussion. The treatment and interviews were completed prior to the start of the COVID-19 pandemic and thus we have no findings on how the pandemic affected our participants. Instead, we are aiming to contextualize our pre-pandemic findings on challenges to care access within the COVID-era healthcare system changes and comment on how these changes may mitigate or worsen barriers to treatment access discussed by participants in our study. We’ve made some minor adjustments to our phrasing in this paragraph to clarify that the study was done prior to 2020.

11. Line 392 – “The lack of HCV knowledge demonstrated by some participants, in conjunction with previous literature demonstrating lack of knowledge as a significant barrier to care.” The authors discuss lack of HCV knowledge as a significant barrier to care. However, the results only discuss lack of knowledge of others (not the participants themselves, but their friends, family…) as a factor contributing to stigma. The results do not discuss participants’ knowledge with regards to HCV (or HCV treatment) and implications, as a result it is not possible to conclude that lack of knowledge of participants was a major barrier.

**Response:** Thank you for your comment. We have adjusted this part of our discussion to better reflect the results and discuss the importance of education as part of the treatment process.

**Reviewer #2’s Comments**

This is an interesting and important research study which highlights the views and lived experience of people who inject drugs in Kenya regarding barriers to treatment for hepatitis C.

The research connected well with the existing literature in terms of barriers around community understanding of HIV and HCV, as well as stigma and changing risk behaviors. The manuscript is well written and justifies itself in light of the increasing prevalence of HCV among PWID in the region. The data analysis & coding process appears to be rigorous.

I noticed that the participant sample skews heavily towards men and I felt this throughout the paper. In light of this, I found myself wanting a little bit more background on gendered roles in Kenya among men and women, at work and with family. As a reader I don’t know anything about men and women in Kenya and how they might be affected differently by stigma, finances, work, and responsibilities around home and family.

Given that this is a paper about the lived experience of at-risk populations, it feels important to perhaps acknowledge and explain the fewer women in the sample, why that might be, and potentially include this as a recommendation for future research to take into account. Are there fewer female PWID in Kenya, are women less able or willing to talk to researchers, or something else? If all of this is outside the scope of the paper, it might be helpful to acknowledge this in the limitations section.

**Response:** Thank you for your feedback. We have added an explanation to the Methods section as to why the study sample included fewer women than men; this was because 1) the parent study was also comprised of mostly men and 2) there are fewer women who inject drugs than men in global and local PWID populations. We have also acknowledged this in the limitations section, and noted the importance of further exploring how gender affects risk behaviors and treatment access.

In terms of the conclusion, it may be difficult to claim the findings can inform improvements to treatments in other LMICs, given the range of countries fitting this definition. While it’s probably reasonable to assume that some of the findings could be applied elsewhere, I think that providing some more detail on similarities between Kenya and other LMICs in terms of the research findings or limiting this claim to Sub-Saharan Africa for example, would strengthen this statement.

**Response:** We have changed the conclusion to suggest that findings may inform interventions in Sub-Saharan Africa instead of all LMICs.

**Reviewer #3’s Comments**

Thank you for the opportunity to review this manuscript regarding HCV care engagement among people with a history of injecting drug use in two regions in Kenya. This is a well written paper and I agree with the authors that this paper provides important insights for improving HCV treatment programs in LMICs. The risk Environment Framework aligns well with the research.

I have made minor comments (mostly editing suggestions) for the authors’ consideration.

1. Abstract – Spell out LMICs in first instance; Mention the theoretical framework which informed the analysis.

**Response:** We have corrected the abstract to include the meaning of LMICs and have mentioned that we used the Rhodes Risk Environment framework.

2. Keywords – consider changing “qualitative” to “qualitative research”

**Response:** We have adjusted the keyword per your suggestion.

3. Line 75 – should “program” be plural?

**Response:** Yes, we have fixed this error to read “programs.”

4. Line 147 – suggest clarifying if this is illicit or prescribed methadone use.

**Response:** We have clarified that the methadone was prescribed.

5. Lines 155-157 – How were these determined/identified?

**Response:** We described our data analysis process through which the themes were determined in the methods section, and have added a few details to provide additional information on the process.

6. Lines 168-170 (quote) – Could the authors provide context on how far this is? It is meaningful for the reader to understand the challenge, e.g., more than one hour by bus.

**Response:** We have added additional context on travel time and fare cost for the reader.

7. Line 202 (quote) – Should this be “pick up the medication”?

**Response:** Yes, we have fixed this error.

8. Line 219 (Mathare) – I had to google this. Perhaps some context for the reader?

**Response:** We have edited the quote to instead read [the methadone clinic] so that it is clearer to the reader what the participant is referring to. We removed the specific clinic name as well to better protect the participant’s privacy.

9. Line 236 (quote) – “When people had that I” – should “had” be ‘heard’?

**Response:** Yes, we have fixed this to read “heard” instead.

10. Lines 238-239 and 250-257 – The two quotes presented align – the stigma and fear of people living with HCV, but also lack of public knowledge that HCV is now treatable (which seems to further compound experiences of stigma among people receiving HCV treatment). Would it be worthwhile to link the two quotes and expand to mention lack of knowledge re: cure?

**Response:** Thank you for this suggestion. We have moved the second quote to link it with the earlier section where participants discuss lack of knowledge about HCV treatment and cure. In introducing the quote, we have also specifically mentioned connections with substance use to connect with the next subtheme.

11. Line 258 (“still”) – delete – this appears to be an extra word inserted.

**Response:** This was a minor typo and has been deleted.

12. Line 262 (“the treatment”) – Are they referring to HCV treatment here or MAT? It isn’t clear how people know he uses drugs if they didn’t know he had hep C.

**Response:** In this case, when the participant says “so the treatment was only from the fact that I was an addict,” he is referring to treatment from others rather than any type of medical treatment. We have adjusted to quote to make this clearer.

13. Line 276 (“treatment”) – Should this be “stigma” instead of “treatment” for reader clarity about what is being referred to?

**Response:** We have adjusted the quote clarification to “stigma” for clarity.

14. Line 328 (“HCV treatment process”) – and challenge of access?

**Response:** We have revised this sentence to better reflect the focus and findings of the paper.

15. Lines 344-346 – Is it being suggested that transport for HCV treatment patients should be provided in Kenya? Is this a feasible consideration?

**Response:** Yes, we are suggesting for interventions to incorporate transportation for patients. We’ve expanded upon this point to note that providing transportation fare stipends/reimbursements (vs. directly providing transportation) has also been shown to help mitigate financial obstacles for patients and may be more logistically feasible.
